# Arginine depletion potentiates standard-of-care chemo-immunotherapy in preclinical models of high-risk neuroblastoma

**DOI:** 10.1186/s13046-025-03502-8

**Published:** 2025-08-14

**Authors:** Kimberley M. Hanssen, Jayne Murray, Ruby Pandher, Stephanie Alfred, Laura D. Gamble, Jennifer Brand, Erin Mosmann, Frances K. Kusuma, Crystal Mak, Adam Kearns, Alvin Kamili, Caroline Atkinson, Alexis Z. Minchaca, Jean Bertoldo, David S. Ziegler, Francis Mussai, Paul N. M. Cheng, Murray D. Norris, Jamie I. Fletcher, Michelle Haber

**Affiliations:** 1 Children’s Cancer Institute Australia, Lowy Cancer Research Centre, UNSW Sydney, Sydney, NSW Australia; 2 School of Clinical Medicine, Faculty of Medicine & Health, UNSW Sydney, Sydney, Australia; 3 Kids Cancer Centre, Sydney Children’s Hospital, Randwick, Australia; 4 Birmingham Children’s Hospital, University of Birmingham, Birmingham, UK; 5 Bio-cancer Treatment International Ltd, Shatin, New Territories, Hong Kong; 6 University of New South Wales Centre for Childhood Cancer Research, UNSW Sydney, Sydney, NSW Australia

**Keywords:** Neuroblastoma, Arginine, Arginase, Metabolism, Preclinical testing, Patient-derived xenografts

## Abstract

**Background:**

Dysregulated amino acid metabolism creates cancer-specific vulnerabilities. Neuroblastoma tumors have dysregulated arginine metabolism that renders them sensitive to systemic arginine deprivation. Arginase therapy has been proposed as a therapeutic approach for neuroblastoma treatment and has a favorable safety profile in pediatric cancer patients, however optimal therapeutic combinations remain unexplored.

**Methods:**

The anti-tumor effects of BCT-100, a pegylated human arginase, were studied in neuroblastoma cell models by metabolite profiling, proteomics, and viability, clonogenicity, and protein translation assays. BCT-100 efficacy was assessed in the Th-*MYCN* transgenic neuroblastoma mouse model and in neuroblastoma cell line and patient-derived xenograft models.

**Results:**

In vitro, depletion of arginine by BCT-100 arrested protein translation and cellular proliferation, with effects on clonogenicity enhanced in combination with standard-of-care chemotherapeutics SN-38/temozolomide and mafosfamide/topotecan. In vivo, BCT-100 treatment spared liver arginine while significantly depleting plasma and tumor arginine in Th-*MYCN* mice, and extended tumor latency (> 100 vs. 45.5 days) in mice pre-emptively treated at weaning. In mice with established tumors, BCT-100 prolonged tumor progression delay when combined with standard-of-care chemo- (> 90 vs. 25 days) or chemo-immuno-therapy (49.5 vs. 35.5 days). Tumor progression delay was also observed in cell line and patient-derived xenografts with BCT-100 treatment, including relapsed/refractory disease models. No increased toxicity was observed with the addition of BCT-100 to established therapies.

**Conclusions:**

The arginase BCT-100 profoundly disrupts neuroblastoma growth in vitro and in vivo, an effect enhanced in combination with standard-of-care chemo-immuno-therapy. Our data supports further assessment of arginine-depleting combination therapies as a new treatment strategy for neuroblastoma.

**Supplementary Information:**

The online version contains supplementary material available at 10.1186/s13046-025-03502-8.

## Background

Dysregulated metabolism is a hallmark of cancer, with metabolic adaptation essential for cancer cells to sustain rapid proliferation, maintain redox homeostasis, and adapt to new microenvironments. These metabolic characteristics distinguish tumor cells from normal cells, making their disruption an attractive, non-genotoxic approach to therapy.

**Fig. 1:**
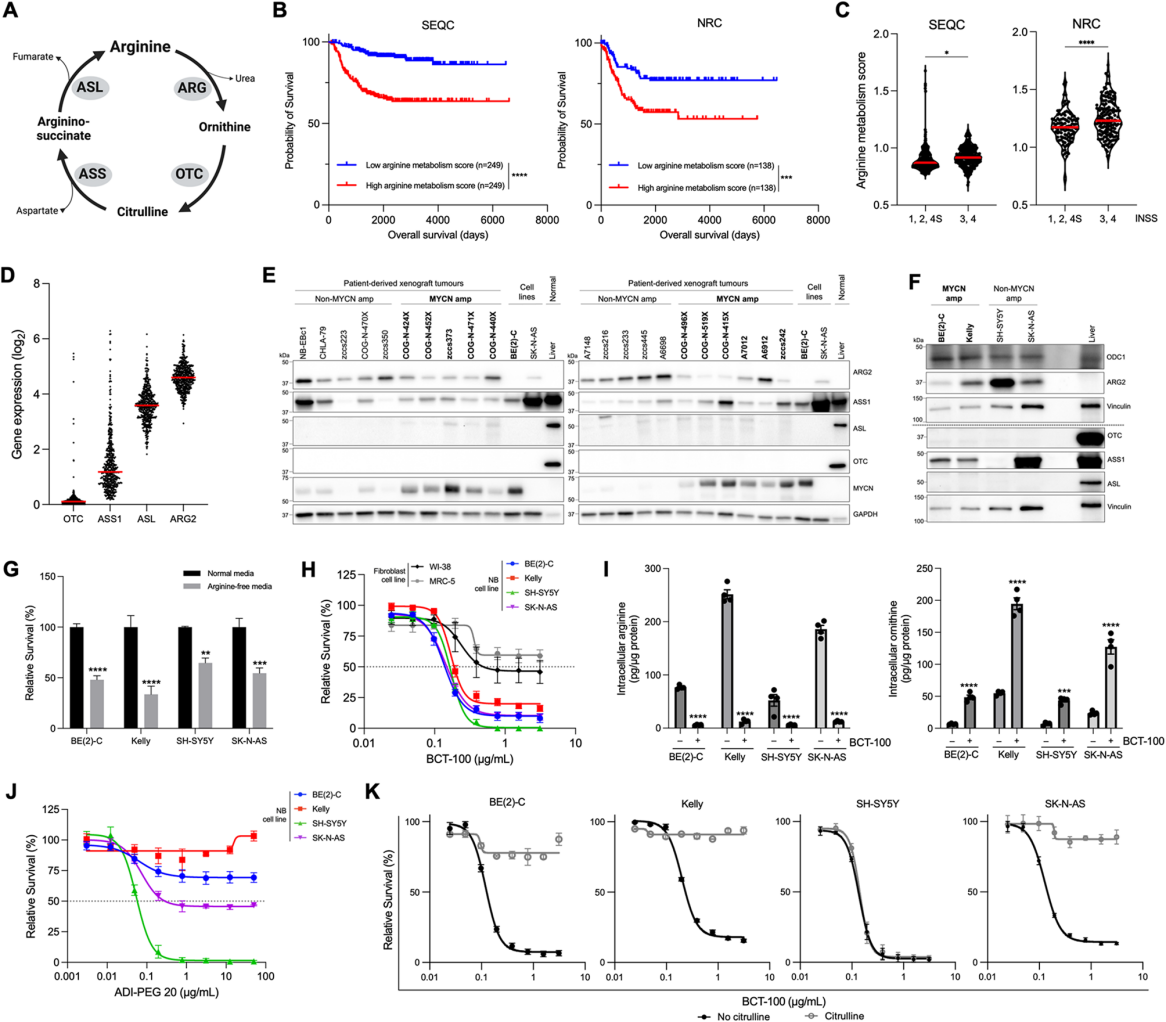
Neuroblastoma tumors rely on extracellular arginine to support proliferation and are susceptible to arginine depletion. (**A**) Schematic of key arginine-cycling enzymes, generated with BioRender.com. (**B–C**) ssGSEA analysis performed on transcriptomic data from primary neuroblastoma tumors (*n* = 498 SEQC dataset, *n* = 283 NRC dataset) from the R2 Genomics and Visualization platform for the KEGG_ARGININE_AND_PROLINE_METABOLISM gene set. (**B**) Kaplan-Meier survival curves of patients with complete clinical data (*n* = 498 SEQC, *n* = 276 NRC) stratified by median ssGSEA score, with two-tailed log-rank test used to test for significance between groups. (**C**) ssGSEA arginine metabolism scores of SEQC and NRC patients stratified by INSS stage 1, 2, 4S vs. more advanced (INSS stage 3, 4) disease. Red line indicates median. Two-sided unpaired t-test. (**D**) Expression of urea cycle enzymes in the SEQC neuroblastoma patient dataset. Red line indicates median. (**E**) Representative western blot of urea cycle enzymes and MYCN in patient-derived neuroblastoma xenografts (PDXs) with GAPDH as a loading control, compared to neuroblastoma cell lines and normal mouse liver (15 µg whole cell lysate for PDXs and cell lines; 7.5 µg for liver). (**F**) Representative western blot of arginine-utilizing (ODC1, ARG2) and arginine-synthesizing (OTC, ASS1, ASL) enzymes in neuroblastoma cell lines with GAPDH as a loading control and normal mouse liver (15 µg whole cell lysate for cell lines and liver). (**G**) Viability of neuroblastoma cells grown in arginine-free media with 10% dialyzed serum or normal media with 10% non-dialyzed serum. Two-way ANOVA with Sidak’s multiple comparisons test. (**H**) Viability of cell lines treated with BCT-100 for 72 h. (**I**) Intracellular concentration of arginine (left) and ornithine (right) in neuroblastoma cell lines 24 h post-treatment with 150 ng/mL BCT-100. Two-way ANOVA with Sidak’s multiple comparisons test. (**J**) Viability of neuroblastoma cell lines treated with BCT-100 ± 1 mM L-citrulline for 72 h. (**K**) Viability of neuroblastoma cell lines treated with ADI-PEG 20 for 72 h. * *P* < 0.05, ** *P* < 0.01, *** *P* < 0.001, **** *P* < 0.0001, ns = not significant. (**G–K**) All data points are mean ± SEM, *n* = 3–4

Arginine is a metabolically versatile amino acid, contributing to proteinogenesis and to the synthesis of nitric oxide, polyamines, nucleotides, proline, glutamate, creatine, urea, and agmatine. Arginine can be synthesized *de novo* from citrulline *via* the successive actions of urea cycle enzymes argininosuccinate synthase 1 (ASS1) and argininosuccinate lyase (ASL), with additional citrulline generated from ornithine *via* ornithine transcarbamylase (OTC) (Fig. 1A). In cancer cells, demand for arginine often surpasses *de novo* synthesis capacity, leading to a reliance on exogenous arginine. Arginine dependence is increased by downregulation of urea cycle enzymes, with ASS1, in particular, frequently aberrant [1]. This adaptation diverts arginine precursors into alternative pathways, including nucleotide biosynthesis, thus conserving energy resources by limiting arginine synthesis in favor of increased uptake [2]. Dependence on exogenous arginine can be exploited therapeutically using arginine deprivation strategies. While arginine dietary restriction in humans only reduces plasma arginine levels by ~ 20–40% [3], arginine-degrading enzymes can achieve therapeutically meaningful decreases in circulating arginine.

Neuroblastoma is the most common extracranial solid tumor of infancy and arises from cells of the primitive sympathetic nervous system [4]. Primary refractory disease, high rates of relapse, and a paucity of therapeutically actionable mutations mean that almost half of all children diagnosed with high-risk neuroblastoma do not survive despite extensive multimodal therapies. New treatment approaches with improved selectivity and safety have the potential to be combined with standard-of-care approaches to achieve better outcomes for these patients.

Neuroblastomas are often arginine auxotrophic, with tumors exhibiting high arginase activity [5] and low ASS1 expression [6]. Consequently, neuroblastoma cell lines are sensitive to arginine-free culture [7]. BCT-100, a pegylated recombinant human arginase 1 [8], metabolizes arginine to ornithine and urea and can rapidly and sustainably deplete plasma arginine levels in adult cancer patients with a favorable safety profile alone or with chemotherapy [9]. In the recent phase I/II *P*egylated recombinant human *A*rginase (BCT-100) in *R*elapsed/refractory cancers of *C*hildren and young adults (PARC) study exploring the safety and activity of BCT-100 in 49 children and young adults with relapsed/refractory cancers, Grade 3 or higher toxicities were rare, and while no patients exhibited a complete response, some patients, including three with high-risk neuroblastoma, maintained stable disease, notable given their confirmed disease progression at time of enrolment [10]. There is currently no preclinical data available for BCT-100 in combination with standard-of-care chemotherapy or immunotherapy for high-risk neuroblastoma, nor with other novel agents. As this knowledge is central to further clinical development of arginine-depleting strategies, we investigated the molecular mechanisms of response to BCT-100 and the clinical potential of BCT-100 combination treatments using patient-derived xenograft (PDX) and genetically-engineered mouse models of neuroblastoma.

## Materials and methods

### Reagents

Reagents included amino acids, temozolomide, chloroquine diphosphate salt, crystal violet, resazurin sodium salt, formic acid, perchloric acid, ethyl acetate, benzoyl chloride, sodium chloride, sodium hydroxide, and acetonitrile (Sigma-Aldrich, St Louis, MO); BCT-100 (Bio-Cancer Treatment International Limited, Hong Kong), ADI-PEG 20 (pegylated arginine deiminase, Polaris Pharmaceuticals Incorporated, San Diego, CA), arginine-free SILAC DMEM and RPMI-1640, and dialyzed fetal calf serum (Thermo Fisher Scientific, Scoresby, VIC, Australia), SN-38 (Cayman Chemical, Ann Arbor, MI), irinotecan (Accord Healthcare, Durham, NC), and mafosfamide sodium salt diastereomer mixture (Toronto Research Chemicals, North York, ON, Canada). Antibodies included: anti-human ganglioside GD2 monoclonal 14G2a (BE0318) and mouse IgG2a isotype control C1.18.4 (BE0085) (Bio X Cell, Lebanon, NH); anti-ARG2 D9J1N (55003), anti-ASS1 D404B (70720), anti-phospho-eIF2α (Ser 51) (9721), anti-eIF2α D7D3 (5324), anti-GAPDH 14C10 (2118), anti-LC3-A/B D3U4C (12741), anti-phospho-S6 (Ser240/244) D68F8 (5364), anti-S6 5G10 (2217), and anti-SLC7A11 D2M7A (12691) (Cell Signaling Technology, Danvers, MA); anti-ASL (ab97370), anti-GAPDH 6C5 (ab8245), anti-ODC1 (ab97395), and anti-OTC (ab244243) (Abcam, Cambridge, UK); anti-ATF4 (10835-1-AP), anti-REDD1 (10638-1-AP), and anti-ASNS (14681-1-AP) (Proteintech, Rosemont, IL); anti-MYCN B8.4.B (sc-53993, Santa Cruz Biotechnology, Dallas, TX); anti-puromycin 12D10 (MABE343; Merck, Darmstadt, Germany); anti-SLC7A1 (PA5-90039, Invitrogen, Waltham, MA); HRP-conjugated goat anti-rabbit IgG (VWR International, Murarrie, QLD, Australia); and HRP-conjugated sheep anti-mouse IgG (GE Healthcare, Rydalmere, NSW, Australia).

### Publicly available data

Transcriptomic and clinical data was obtained through the R2 Genomics Analysis and Visualization Platform (http://r2.amc.nl) for two independent primary neuroblastoma tumor cohorts: *n* = 498 from the Sequencing Quality Control (SEQC) consortium (GSE62564) [11] and *n* = 283 (*n* = 276 with complete clinical data) from the Neuroblastoma Research Consortium (NRC) (GSE85047) [12]. Single-sample gene set enrichment analysis (ssGSEA) used the *gsva* R package (v.1.48.3) [13] with default parameters to evaluate the enrichment score for each tumor using the MSigDB ‘KEGG_ARGININE_AND_PROLINE_METABOLISM’ gene set. Patients were dichotomized to high- or low-arginine metabolism groups at the median ssGSEA score. Kaplan-Meier analysis with a two-sided log-rank test was performed to determine the statistical difference in overall survival between groups.

Gene expression data for 917 cell lines from the Cancer Cell Line Encyclopedia (CCLE; GEO accession number GSE36133) [14] was obtained through the R2 platform.

### Cell lines and culture

The identity of all cell lines, including SK-N-BE(2)-C (BE(2)-C) and SH-SY5Y (Professor June Biedler, Memorial Sloan-Kettering Cancer Center, New York, NY), Kelly and SK-N-AS (European Collection of Cell Cultures), and WI-38 and MRC5 (American Type Culture Collection, Manassas, VA) was confirmed by STR profiling (Garvan Medical Institute, Sydney, Australia) and each was verified negative for mycoplasma. Each was cultured in media (DMEM: BE(2)-C, SH-SY5Y; RPMI-1640: Kelly; MEMα: WI-38, MRC5) containing 10% fetal bovine serum (Thermotrace Nobel Park, VIC, Australia) and incubated at 37 °C and 5% CO_2_ in a humidified environment.

### Cytotoxicity and clonogenic assays

Cells were seeded in triplicate into 96-well plates at 5 × 10^3^ (BE(2)-C), 6 × 10^3^ (SH-SY5Y, SK-N-AS), or 8 × 10^3^ (Kelly) cells/well, adhered overnight, then exposed to BCT-100, ADI-PEG 20, chloroquine, or arginine-free media (containing 10% dialyzed serum). BCT-100 rescue experiments were supplemented with specific amino acids. Viability was determined 72 h post-exposure using a resazurin-based assay [15] (570/595 nm; Benchmark Plus plate reader, Bio-Rad, Hercules, CA).

Clonogenic assays were in triplicate in 6-well plates at 300 (BE(2)-C), 250 (Kelly), 600 (SH-SY5Y), or 700 (SK-N-AS) cells/well, adhered overnight, then exposed to BCT-100, chemotherapy, or both in a fixed ratio for 72 h. Colonies were stained 11–14 days post-plating with 0.5% crystal violet in 50% methanol, imaged using the Gel Doc XR+ system (Bio-Rad) and quantified using ImageJ [16].

Cells for drug washout assays were seeded in 6-well plates at 2 × 10^4^ cells/well, adhered overnight, exposed to 200 ng/mL BCT-100 for 3–15 days, and counted by trypan blue assay every 3 days to determine recovery following BCT-100 washout and arginine replenishment.

### PDX models

Neuroblastoma PDX models were obtained from the Childhood Cancer Repository, Texas Tech University Health Sciences Center (Lubbock, Texas) or established as part of the ZERO Childhood Cancer Study using fresh biopsy or bone marrow from high-risk neuroblastoma patients [17–19]. For ex vivo drug assays, dissociated PDX cells were seeded into 96-well plates in IMDM media with 20% fetal bovine serum and 1× insulin-transferrin-selenium and incubated under reduced oxygen conditions (5% O_2_) for 72 h prior to exposure to BCT-100 for an additional 72 h. Cells were then incubated with resazurin overnight and viability determined as above.

### Protein isolation and Western blotting

Whole cell lysates were isolated from tumor, mouse liver and cell pellets as previously described [20], and protein concentrations determined by BCA assay (ThermoFisher Scientific). Lysates were resolved by SDS-PAGE (Bio-Rad), transferred to PVDF membrane (Merck), blocked and incubated overnight at 4 °C with primary antibodies (1:500–1000 dilution), then with secondary antibodies (1:2000 dilution) for 1 h at room temperature. Chemiluminescent signals were visualized using Clarity ECL Western Substrate and a ChemiDoc MP Imaging System (Bio-Rad). Signal was normalized to GAPDH and, where applicable, to non-phosphorylated total protein and are shown relative to control treated conditions, as estimated by densitometry using ImageJ software [16].

### Protein expression and puromycin incorporation

Cells were seeded in 6-well plates at 2 × 10^5^ (BE(2)-C, Kelly), 2.4 × 10^5^ (SH-SY5Y), or 1.8 × 10^5^ (SK-N-AS) cells/well, adhered overnight, then exposed to BCT-100. Cell lysates collected 72 h post-exposure were subjected to SDS-PAGE and western blotting. For puromycin incorporation, cells were exposed to media containing 10 µg/mL puromycin 10 min prior to collection and puromycin incorporation evaluated by western blot [21].

### Autophagy

Cells were seeded at 3 × 10^5^ cells/well in 6-well plates, adhered overnight, then exposed to 150 ng/mL BCT-100 for 48 h. During the last 4 h, 100 µM chloroquine was added. Cells were collected, protein extracted, and subjected to SDS-PAGE and western blotting using an anti-LC3 A/B antibody [22].

### Quantitation of arginine and ornithine

Arginine and ornithine were quantitated by LC-MS/MS from tumors or cell pellets following pre-column benzoyl chloride derivatization [23]. Samples were homogenized (tissue) or scraped from a culture dish (1% formic acid/PBS), deproteinized (10% perchloric acid), and centrifuged (13,400 × *g*, 5 min, 4 °C). Supernatant was neutralized (2 N NaOH), mixed with 4% benzoyl chloride in acetonitrile (37 °C, 30 min, 1200 rpm) and mixed with saturated NaCl and ethyl acetate. Standards were prepared in PBS with 1% formic acid and subjected to the same derivatization steps.

LC-MS/MS was performed on a ThermoFisher U3000 System (10 µL samples) with separation on an XBridge BEH C18 2.1 × 50 mm UHPLC column (Waters, Milford, MA) at 300 µL/min using a 10 min linear acetonitrile gradient with eluate directed to a Quantum Access mass spectrometer (ThermoFisher Scientific) with analysis in the positive ion mode. Arginine was detected using SRM transition 279/105 and ornithine using the transition 341/105. Data was processed and chromatograms integrated automatically using XCalibur software and normalized to protein concentration.

### Proteomics analyses

Cells (0.9 × 10^5^ BE(2)-C, 1.0 × 10^5^ SH-SY5Y) were seeded into 60 mm dishes, adhered overnight, then exposed to 170 ng/mL (SH-SY5Y) or 200 ng/mL (BE(2)-C) BCT-100 for 72 h. Cells were lysed in RIPA buffer containing protease and phosphatase inhibitors, protein concentrations determined by BCA assay, and samples prepared and used for mass spectrometry-based label-free proteomics with a 90 min gradient as described [24]. Protein identification and label-free quantification (LFQ) were analyzed using MaxQuant (v.2.1.3.0) and the integrated Andromeda search engine algorithm with default parameters [25]. Perseus (v.2.0.7.0) was used for subsequent data processing [26]. For differential expression analyses, missing LFQ values were imputed from normal distribution and statistical comparisons performed using a two-sided *t*-test with a permutation-based FDR of 0.05. For pathway enrichment analysis, proteins were ranked by their log_2_ fold-change and used with the *fgsea* R package (v.1.28.0) with MSigDB Hallmark gene sets [27].

### Th-*MYCN* Transgenic mouse model

Th-*MYCN* transgenic mice [28] were maintained as previously described [29]. Male and female Th-*MYCN*^+/+^ mice were randomized into treatment groups at small palpable (~ 5 mm) abdominal tumor or at 3 weeks old in the prophylaxis setting, and were humanely euthanized at 10 mm abdominal tumor, signs of a thoracic tumor (hind limb paralysis or labored breathing), or in the absence of relapse at 20 weeks old. For prophylaxis studies, mice were treated with 60 mg/kg BCT-100 intraperitoneally (IP) two-times weekly (i.e. dosing every 3–4 days) or four-time weekly (i.e. dosing every other day). For combination chemotherapy, mice were treated with 2 mg/kg/day irinotecan and 5 mg/kg/day temozolomide IP for 5 days with or without 60 mg/kg IP BCT-100 (four-times weekly until endpoint). For combination immunotherapy, mice were treated with 2 mg/kg/day irinotecan and 5 mg/kg/day temozolomide IP on days 1 and 2, 60 mg/kg IP BCT-100 four-times weekly until endpoint and 15 µg anti-GD2 or isotype control on days 1 and 5, a dose which provides clinically equivalent peak plasma levels in mice (~ 8 µg/mL) [30, 31]. The chemoimmunotherapy schedule allows relapse within 50 days, suitable for assessing addition of new agents [32].

For short timepoint assessment of plasma and tumor arginine, mice were treated with IP saline vehicle or 60 mg/kg BCT-100 every other day then humanely euthanized 24 h after the second or fourth dose.

Plasma and tissue samples were collected at the specified short timepoints or otherwise at endpoint, snap-frozen and stored at − 80 °C for downstream analyses.

### Cell line and PDX xenograft models

Tumor cells (1 × 10^6^ SH-SY5Y or PDX cells) were engrafted in 50% growth factor-reduced Matrigel (Corning, NY) subcutaneously in the dorsal flank of female mice: 5–6-week-old Balb/c nude mice (BALB/*c*-*Foxn1*^*nu*^*/*Ozarc; Ozgene ARC, Perth, WA, Australia) for SH-SY5Y and 6–8-week-old NSG mice (NOD.Cg-Prkdc^*scid*^Il2rg^tm1Wjl^/SzJ; Australian BioResources, Moss Vale, NSW, Australia) for PDXs. Tumor size ([length × width × depth]/2) was measured every other day and mice randomized to treatment groups at 100 mm^3^ tumor. Mice were treated with irinotecan/temozolomide/BCT-100 as above. The maximal tumor volume permitted by the ethics approval was 1000 mm^3^ and mice were humanely killed upon reaching this tumor volume. Plasma and tissue samples were collected at endpoint, snap-frozen, and stored at − 80 °C for downstream analyses.

### Statistics

Curve fitting, statistical analysis, and data visualization used GraphPad Prism 9 (GraphPad Software, La Jolla, CA). Dose-response curves were fitted with a four-parameter logistic model by non-linear regression. Data are presented as means ± standard error (SEM) of at least three independent experiments. Statistical differences were assessed by two-sided unpaired Student’s *t*-test for two groups and by one- or two-way ANOVA for three or more groups. In cases where *F* achieved statistical significance, Dunnett’s or Sidak test for multiple comparisons were applied. Kaplan-Meier curves were generated from survival data and two-sided log-rank tests performed to determine statistical differences in survival between treatment groups. P values < 0.05 were considered statistically significant.

## Results

### Neuroblastomas rely on extracellular arginine

To investigate the clinical significance of arginine metabolism, we first performed ssGSEA for the KEGG_ARGININE_AND_PROLINE_METABOLISM gene set on transcriptomic data from the SEQC and NRC primary neuroblastoma cohorts. Patients with above-median arginine metabolism scores had significantly shorter overall survival in both cohorts (Fig. 1B) and tumors from patients with advanced INSS stages also had higher median arginine metabolism scores (Fig. 1C). Expression of key *de novo* arginine synthesis enzymes (OTC, ASS1) was low in most patient tumors (SEQC; Fig. 1D) and in most samples from a panel of 21 PDX models and four cell lines, when compared to normal mouse liver control (Fig. 1E–F). Next, we evaluated the contribution of exogenous arginine to neuroblastoma proliferation in four neuroblastoma cell lines (MYCN-amplified BE(2)-C and Kelly; non-MYCN-amplified SH-SY5Y and SK-N-AS) by culture in standard or arginine-free media. Each line had significantly decreased viability under arginine-depleted conditions (Fig. 1G). Extracellular arginine is thus needed to supplement *de novo* synthesis to maintain neuroblastoma cell growth and viability.

### Neuroblastoma cells are susceptible to arginine depletion by BCT-100

We then tested whether arginine-depleting therapy reduces neuroblastoma viability. Exposure of neuroblastoma cell lines to BCT-100 caused a dose-dependent reduction in viability (Fig. 1H) with the IC_50_ ≥ 2-fold lower than for fibroblast cell lines WI-38 and MRC5. Arginase activity was confirmed by LC-MS/MS with a > 8-fold reduction in intracellular arginine and a concomitant > 6-fold increase in intracellular ornithine, a BCT-100 product, within 24 h of exposure (Fig. 1I). We confirmed that the anti-proliferative effect of BCT-100 was due to arginine depletion by supplementing the media with citrulline (Fig. 1J), which rescued the anti-proliferative effect of BCT-100 in the ASS1-expressing BE(2)-C, Kelly, and SK-N-AS cells but not the ASS1-negative SH-SY5Y cells. Consistent with this finding, the related arginine hydrolase, ADI-PEG 20, which metabolizes arginine to citrulline and ammonia, was only effective against the ASS1-negative SH-SY5Y cells which are unable to utilize citrulline to re-synthesize arginine (Fig. 1K). BCT-100 is thus active against neuroblastoma cells irrespective of ASS1 expression.

### BCT-100 reduces global protein translation by inducing the ATF4/REDD1 stress response and inhibiting mTORC1 signaling

**Fig. 2:**
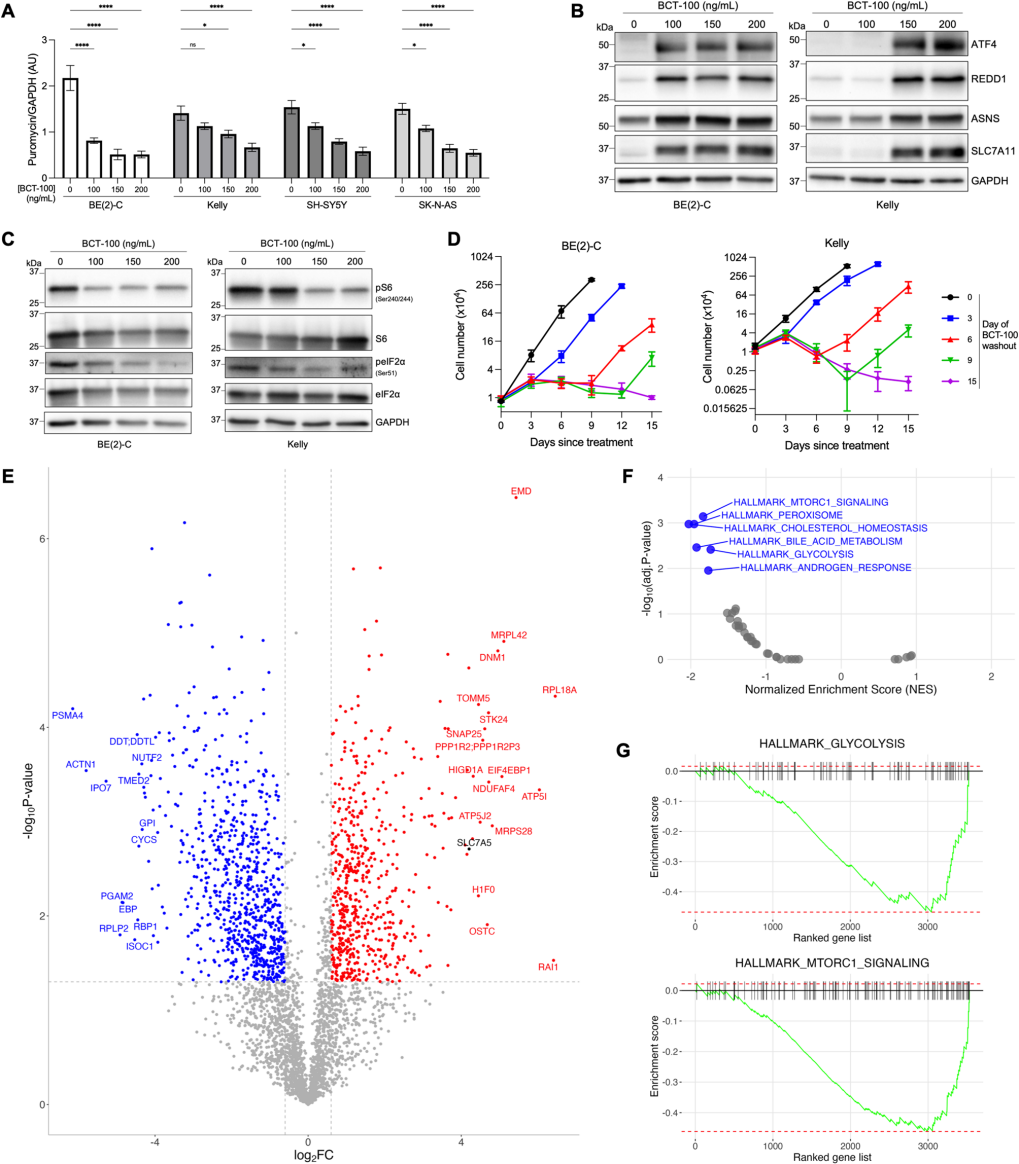
BCT-100 reduces global protein translation, activates cell stress response, and suppresses cell proliferation. **(A)** Densitometric quantitation of puromycin incorporation, adjusted for the level of GAPDH, from western blots of BE(2)-C, Kelly, SH-SY5Y, and SK-N-AS whole cell lysates 72 h post-exposure to BCT-100. **(B)** Representative western blot of the stress response protein ATF4 and downstream pathway targets REDD1, ASNS and SLC7A11 in whole cell lysates of BE(2)-C and Kelly cells at 72 h post-exposure to BCT-100. **(C)** Representative western blot of the ISR regulator eIF2α, the mTORC1 regulated S6, and their phosphorylation states in whole cell lysates of BE(2)-C and Kelly cells at 72 h post-exposure to BCT-100. **(A–C)** All western blots are from 20 μg whole cell lysate and similar results were obtained in two or more additional independent experimental replicates, each using independent lysate preparations and quantified by densitometry (Supplementary Figure S1). **(D)** Number of neuroblastoma cells at 0, 3, 6, 9, 12, or 15 days post-exposure to 150 ng/mL BCT-100 with or without removal of BCT-100 by media change (‘washout’). **(E)** Volcano plot of differentially expressed proteins comparing BCT-100 exposed vs non-exposed BE(2)-C cells. **(F–G)** Significantly altered Hallmark gene sets from fGSEA pathway enrichment analysis (adjusted P-value <0.05). All data points are mean ± SEM, *n* = 3.

As amino acid insufficiency can inhibit protein synthesis, we examined global protein translation rates by puromycin incorporation following BCT-100 exposure. We found puromycin incorporation decreased across the neuroblastoma cell lines with increasing BCT-100 dose (Fig. 2A, Supplementary Figure S1A). Next, we sought to clarify which of the interconnected stress-adaptive pathways was responsible for the impact of BCT-100 on protein translation. Arginine starvation can induce the integrated stress response (ISR) by stimulating stress kinases which, via phosphorylation of eIF2α, globally attenuate Cap-dependent translation while concomitantly upregulating translation of select stress-response genes [33]. The ISR transcription factor ATF4 was strongly induced by BCT-100, along with downstream targets REDD1, ASNS and SLC7A11 (Fig. 2B, Supplementary Figures S1B–F), however we observed decreased phosphorylation of eIF2α (Fig. 2C, Supplementary Figure S1G–H). Protein translation can also be downregulated when mTORC1 activity is inhibited by arginine depletion, mediated by reduced phosphorylation of the 40S ribosomal subunit ribosomal protein S6 by the effector S6K1, which normally positively regulates cap-dependent translation initiation [34]. Phosphorylation of S6 was decreased by BCT-100 (Fig. 2C, Supplementary Figures S1G, I), and thus BCT-100 appears to reduce global protein translation through multiple mechanisms, including ISR induction and mTORC1 inhibition.

ASNS upregulation caused by arginine depletion leads to cell death in MDA-MB-231 breast cancer cells by exhausting intracellular aspartate and disrupting the malate-aspartate shuttle needed for mitochondrial function [35]. Supplementation of supraphysiological concentrations of the ASNS substrate aspartic acid, however, did not significantly rescue the viability of the four neuroblastoma cell lines exposed to BCT-100 (Supplementary Figure S2), marking the response of neuroblastoma cells to arginine depletion as distinct from that of other cancer models despite similar induction of ASNS under arginine starved conditions.

Given the suppression of protein translation, we next investigated the impact of BCT-100 on cell proliferation. Exposure to BCT-100 completely abrogated cell proliferation in the four neuroblastoma cell lines and showed a decline in cell number indicative of cell death after 6–9 days of continuous BCT-100 exposure in all lines except BE(2)-C (Fig. 2D, Supplementary Figure S3A). However, cell proliferation rapidly resumed upon removal of BCT-100 and replenishment of extracellular arginine by media change. Thus, in neuroblastoma cells, arginine depletion reversibly abrogates cell proliferation but induces cell death in only a subpopulation of cells as a monotherapy.

### Glycolysis and lipid metabolism are downregulated by BCT-100

To better understand the mechanisms underlying growth suppression with arginine depletion, proteomics analysis was conducted on BE(2)-C and SH-SY5Y cells following 72 h BCT-100 exposure. Significant changes in protein expression were observed (BE(2)-C: 595 upregulated, 800 downregulated proteins; SH-SY5Y: 307 upregulated, 466 downregulated proteins; *p* < 0.05,|log_2_FC|>0.6) (Fig. 2E, Supplementary Figure S3B). Of note, the citrulline importer SLC7A5, recently found to be required for cell growth under arginine-depletion [36], was significantly upregulated in both cell lines. Pathway enrichment analysis found the metabolic hallmarks of glycolysis and cholesterol/fatty acid metabolism were consistently downregulated (Fig. 2F–G, Supplementary Figure S3C–D), as well as downregulated mTORC1 signaling in BE(2)-C cells, demonstrating that arginine depletion broadly and severely impedes the metabolic activity of neuroblastoma cells.

### BCT-100 extends survival of a Transgenic mouse model of neuroblastoma

**Fig. 3:**
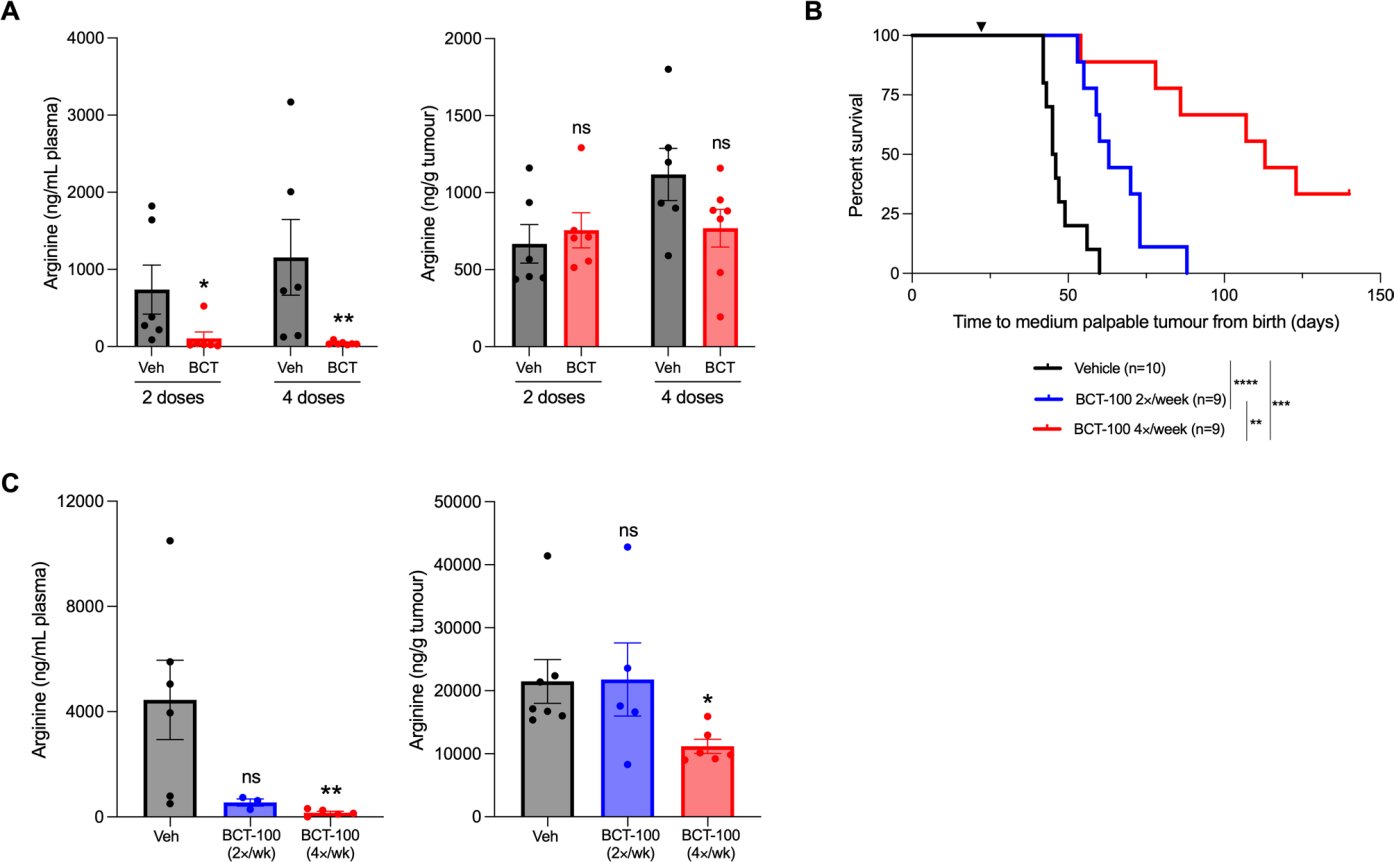
BCT-100 reduces plasma/tumor arginine and extends survival as a single agent in Th-*MYCN* mice. (**A**) Concentration of arginine in plasma (left) or tumor tissue (right) of Th-*MYCN* mice treated with 60 mg/kg BCT-100 or saline vehicle 24 h after receiving a second or fourth dose as determined by LC-MS/MS. Mann Whitney test was used to test the significance between BCT-100 and vehicle treated groups for each 2 and 4 doses. (**B**) Kaplan-Meier survival curves showing the survival of Th-*MYCN* transgenic mice after prophylactic treatment with 60 mg/kg two- or four-times weekly from time of weaning (3 weeks of age), before detectable tumor formation. The black arrow indicates commencement of treatment. Two-tailed log-rank tests were used to test for significance between treatment groups. (**C**) Concentration of arginine in plasma (left) or tumor tissue (right) of Th-*MYCN* mice at endpoint from (**B**) as determined by LC-MS/MS. Kruskal-Wallace test with Dunn’s multiple comparisons test was used to test for significance between BCT-100 treatment groups and vehicle control. * *P* < 0.05, ** *P* < 0.01, *** *P* < 0.001, **** *P* < 0.0001, ns = not significant. All data points are mean ± SEM, *n* = 3–7

To test the ability of BCT-100 to deplete circulating arginine in the context of neuroblastoma, we first treated Th-*MYCN* mice, upon detection of a small palpable tumor, with 2 or 4 doses of BCT-100 and examined plasma and tumor arginine levels 24 h following the last dose. Plasma arginine was significantly depleted after two doses of BCT-100, while steady-state intra-tumoral arginine levels were not significantly reduced following four doses (Fig. 3A).

Next, we tested the impact of BCT-100 on tumor latency and progression. Prophylactic treatment of Th-*MYCN* mice with BCT-100 from time of weaning (i.e. from before the development of detectable tumors) significantly extended survival (Fig. 3B). Mice survived a median of 45.5 days from birth with vehicle, 63 days with 2×/week BCT-100 and beyond 100 days with 4×/week dosing, including 3 of 9 mice with no detectable tumor by study endpoint of 120 days. At endpoint, arginine depletion was maintained in plasma while significant arginine depletion was only achieved in tumors from mice dosed 4×/week (Fig. 3C).

### BCT-100 exposure influences urea cycle enzyme expression and autophagy but not arginine transport

BCT-100 monotherapy substantially prolonged survival of Th-*MYCN* mice, but despite sustained plasma arginine depletion, most animals ultimately succumbed to their tumors by study endpoint. We thus investigated mechanisms that may replenish arginine or promote cell survival under amino acid limiting conditions.

**Fig. 4:**
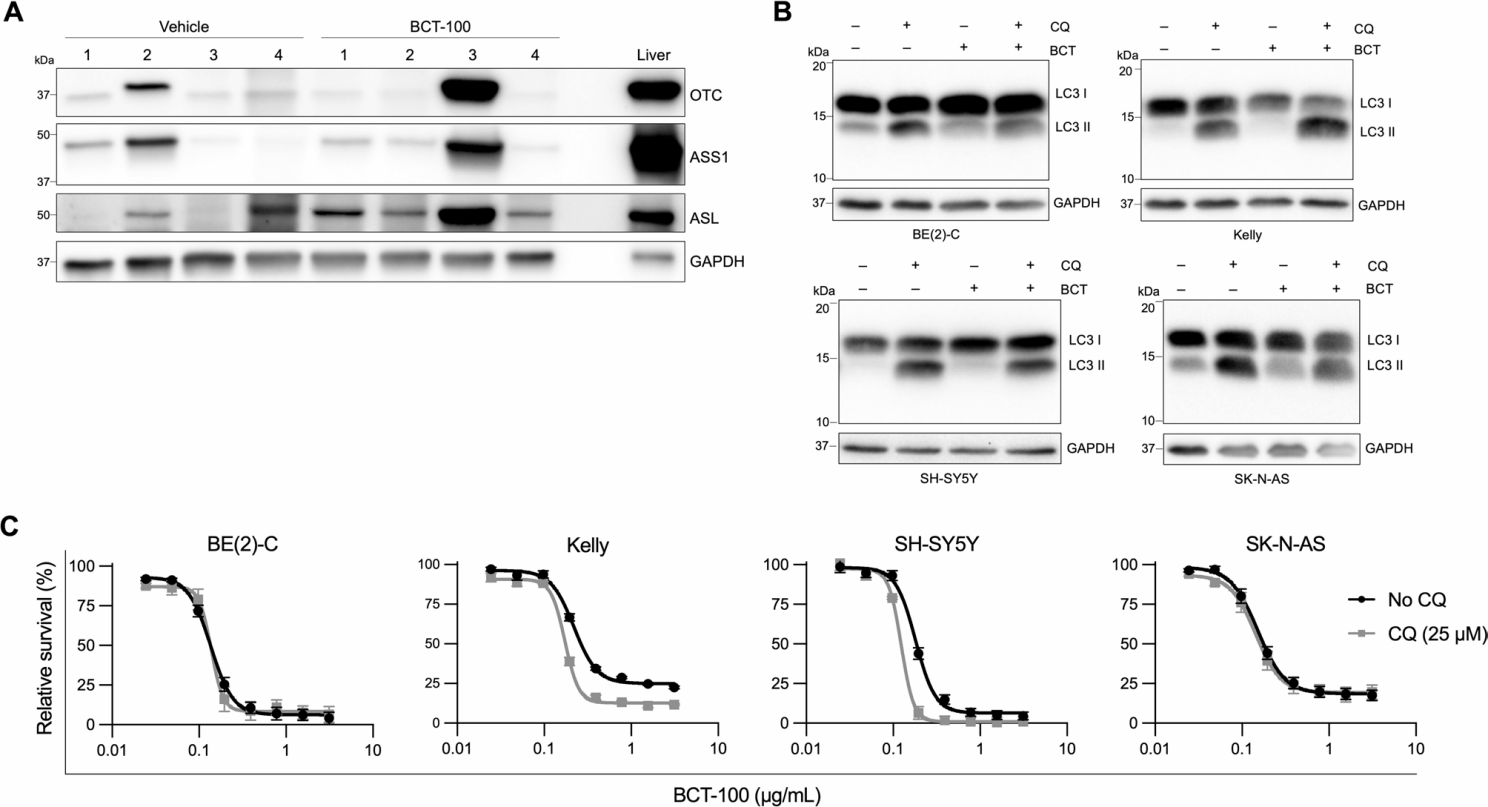
BCT-100 upregulates urea cycle enzymes and induces autophagy in some but not all cell lines/tumors. (**A**) Representative western blot of urea cycle enzymes (OTC, ASS1, ASL) in mouse tumors at endpoint following treatment with BCT-100 four-times weekly from time of weaning (Fig. 3B), with GAPDH and normal mouse liver as the loading and positive controls respectively (20 µg whole cell lysate). (**B**) Representative western blots of LC3 I and II levels in whole cell lysates of neuroblastoma cells exposed to BCT-100 for 48 h with or without 100 µM chloroquine for the last 4 h, with GAPDH as a loading control (20 µg whole cell lysate). Similar western blot results were obtained in two additional independent experimental replicates. (**C**) Viability of neuroblastoma cell lines exposed to increasing concentrations of BCT-100 with or without 25 µM chloroquine (CQ) for 72 h. All data points are mean ± SEM, *n* = 3

We first examined the expression of urea cycle enzymes in BCT-100-treated Th-*MYCN* mouse tumors at endpoint. One of four treated tumors assessed showed urea cycle enzyme upregulation compared to vehicle controls at endpoint (Fig. 4A) and had the highest arginine level of endpoint tumors from BCT-100 treated mice (Fig. 3C). In contrast, urea cycle enzyme expression was unaltered following 72 h BCT-100 exposure in neuroblastoma cell lines (Supplementary Figure S4A). Expression of SLC7A1/CAT-1, the predominant arginine importer in primary neuroblastoma tumors (Supplementary Figure S4B) and in the 4 selected neuroblastoma cell lines (Supplementary Figure S4C), was variably affected by BCT-100 exposure in a cell line- and concentration-dependent manner in vitro (Supplementary Figure S4D–E). We next assessed autophagy, a mechanism by which cells can temporarily recycle cell components to obtain arginine for synthesis of critical proteins and metabolites. BCT-100 increased autophagic flux, measured by LC3-II accumulation following co-exposure to the autophagy inhibitor chloroquine, in Kelly but not BE(2)-C, SH-SY5Y, or SK-N-AS cells (Fig. 4B, increase compared to chloroquine alone), while autophagy inhibition moderately enhanced BCT-100 sensitivity in Kelly and SH-SY5Y, but not BE(2)-C or SK-N-AS cells (Fig. 4C). Arginine replenishment by re-expression of urea cycle enzymes and autophagy are thus possible mechanisms that impact on BCT-100 sensitivity.

### BCT-100 enhances chemo- and immunotherapy in Th-*MYCN* mice

**Fig. 5:**
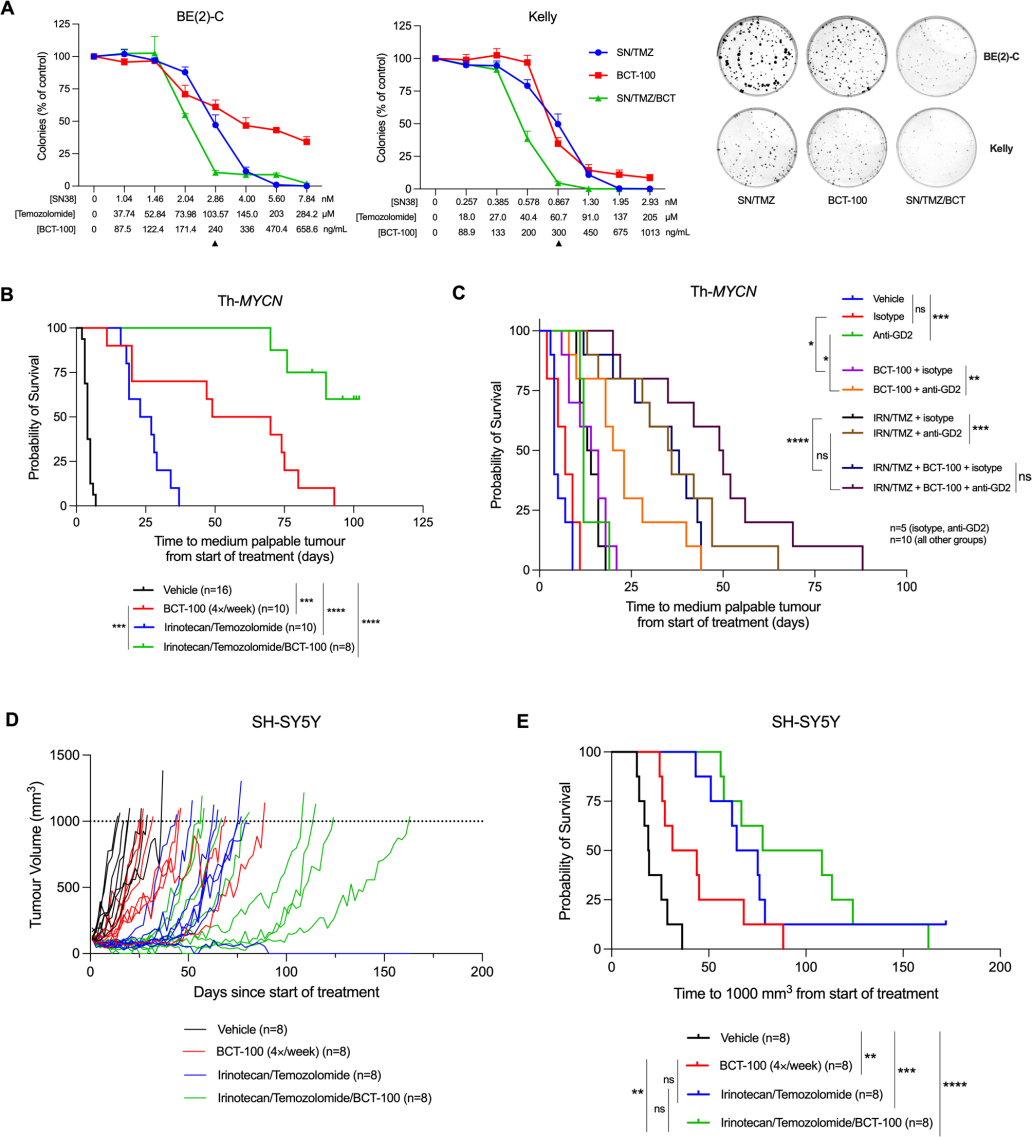
BCT-100 enhances the anti-cancer efficacy of standard-of-care chemotherapeutics in vitro and in vivo. (**A**) Clonogenic capacity of BE(2)-C and Kelly cells exposed to BCT-100, SN-38/temozolomide (SN/TMZ), or a combination of SN/TMZ and BCT-100 (SN/TMZ/BCT) in a fixed ratio for 72 h. Representative images of colonies are shown to the right, with the black arrow indicating the dose represented in the images. (**B**) Kaplan-Meier survival curves showing the survival of Th-*MYCN* transgenic mice after treatment with irinotecan (2 mg/kg/day) and temozolomide (5 mg/kg/day) for 5 days, 60 mg/kg BCT-100 four-times weekly, or a combination of both from detection of a small palpable tumor. (**C**) Kaplan-Meier survival curves showing the survival of Th-*MYCN* transgenic mice after treatment with 60 mg/kg BCT-100 four-times weekly, irinotecan (2 mg/kg/day)/temozolomide (5 mg/kg/day) for 2 days, or irinotecan/temozolomide/BCT-100 with two doses of 15 µg anti-GD2 antibody or IgGa isotype, from detection of a small palpable tumor. (**D**) Tumor growth curves and (**E**) Kaplan Meier survival curve of Balb/c nude mice subcutaneously engrafted with SH-SY5Y cells after treatment with 60 mg/kg BCT-100 four-times weekly until endpoint, irinotecan (2 mg/kg/day)/temozolomide (5 mg/kg/day) for 5 days, or a combination of both from when tumors reached 100 mm^3^. Two-tailed log-rank tests were used to test for significance between treatment groups for the Kaplan-Meier survival curves. * *P* < 0.05, ** *P* < 0.01, ***P < 0.001, **** *P* < 0.0001, ns = not significant. All data points are mean ± SEM, *n* = 3–5

To understand clinical potential, we modelled response to BCT-100 in combination with standard-of-care chemotherapy and chemoimmunotherapy. In clonogenic assays, BCT-100 potentiated SN-38/temozolomide activity (modelling irinotecan/temozolomide) in BE(2)-C and Kelly (Fig. 5A) and to a lesser extent in SH-SY5Y and SK-N-AS (Supplementary Figure S5A). Similar potentiation was observed with mafosfamide/topotecan, modelling cyclophosphamide/topotecan (Supplementary Figure S5B).

In Th-*MYCN* mice, BCT-100/irinotecan/temozolomide extended median survival to > 90 days since start of treatment with no detectable tumor in 5 of 8 of mice at the study endpoint, compared to vehicle (4.0 days), BCT-100 (59.5 days), or irinotecan/temozolomide (25.0 days) (Fig. 5B). No dose-limiting toxicities were observed in the triple combination. Plasma arginine depletion was confirmed at endpoint in BCT-100-treated mice, although tumor arginine depletion did not reach statistical significance due to high variability between animals (Supplementary Fig. 5C). ASS1 upregulation was observed at endpoint in 1 of 6 BCT-100 treated tumors assessed (Supplementary Figure S5D).

Next, we tested whether BCT-100 treatment potentiates the anti-tumor effect of anti-GD2 antibody, a frontline immunotherapy for neuroblastoma, in the presence or absence of chemotherapy. At reduced doses, irinotecan/temozolomide and anti-GD2 antibody prolonged survival of Th-*MYCN* mice to a similar extent as BCT-100 monotherapy (median survival 13.5, 12, and 15 days respectively) (Fig. 5C). The addition of anti-GD2 therapy to BCT-100 or irinotecan/temozolomide significantly improved median survival compared to combination with isotype control (15 days BCT-100/isotype vs. 21.5 days BCT-100/anti-GD2; 13.5 days IRN/TMZ/isotype vs. 35.5 days IRN/TMZ/anti-GD2). The combination of BCT-100/irinotecan/temozolomide/anti-GD2 further extended median survival to 49.5 days. BCT-100 thus potentiates both standard-of-care chemotherapy and immunotherapy, prolonging survival even when administered as a third agent on top of combined chemo-immuno-therapy without increasing toxicity.

### BCT-100 enhances chemotherapy in human neuroblastoma xenografts

We next extended our combination studies to human tumors using xenograft models. In SH-SY5Y cell line xenografts in Balb/c nude mice, BCT-100 delayed tumor progression, increasing median survival from 19.1 to 37.6 days (Fig. 5D–E). The combination of BCT-100 with standard-of-care irinotecan/temozolomide prolonged median survival (93 days) compared to chemotherapy alone (69.8 days) without dose-limiting toxicities, although this did not reach statistical significance.

**Fig. 6:**
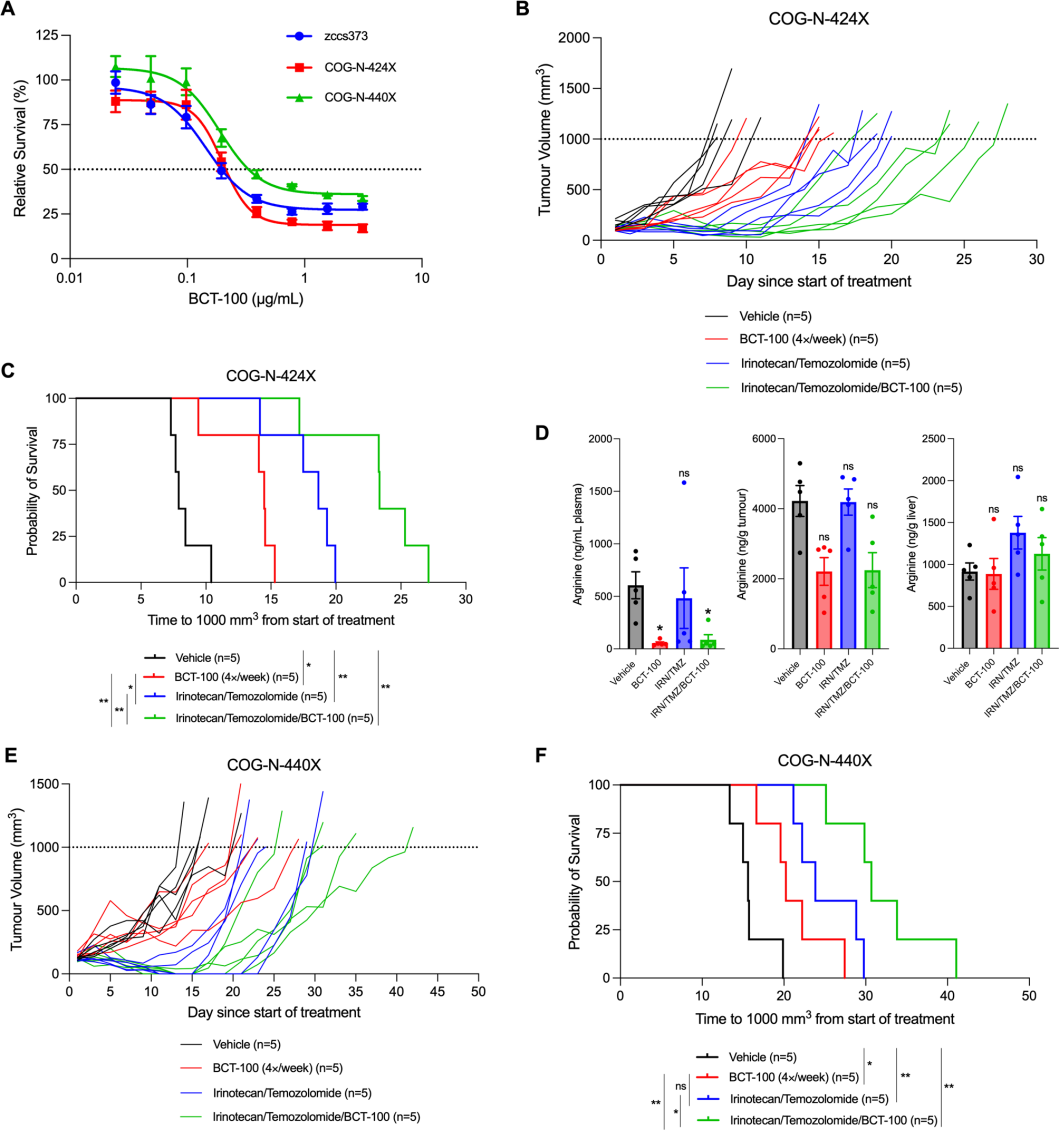
BCT-100 is effective in combination with standard-of-care chemotherapeutics against neuroblastoma PDXs in vivo. (**A**) Viability of ex vivo neuroblastoma PDX cells exposed to increasing concentrations of BCT-100 for 72 h. (**B**) Tumor growth and (**C**) Kaplan-Meier survival curves of NSG mice bearing COG-N-424X xenografts after treatment with 60 mg/kg BCT-100 four-times weekly until endpoint, irinotecan (2 mg/kg/day)/temozolomide (5 mg/kg/day) for 5 days, or a combination of both from when tumors reached 100 mm^3^. Two-tailed log-rank tests were used to test for significance between treatment groups for the Kaplan-Meier survival curves. (**D**) Concentration of arginine in plasma (left), tumor tissue (middle), or liver tissue (right) from COG-N-424X engrafted mice from (**B–C**) at endpoint as determined by LC-MS/MS. Kruskal-Wallace test with Dunn’s multiple comparisons test was used to test for significance between treatment groups and vehicle control. (**E**) Tumor growth and (**F**) Kaplan-Meier survival curves of NSG mice bearing COG-N-440X xenografts after treatment with 60 mg/kg BCT-100 four-times weekly until endpoint, irinotecan (2 mg/kg/day)/temozolomide (5 mg/kg/day) for 5 days, or a combination of both from when tumors reached 100 mm^3^. Two-tailed log-rank tests were used to test for significance between treatment groups for the Kaplan-Meier survival curves. * *P* < 0.05, ** *P* < 0.001, ***P < 0.001, **** *P* < 0.0001, ns = not significant. All data points are mean ± SEM, (**A**) *n* = 3–8, (**D**) *n* = 5

Extending studies to PDX models, cells dissociated from 3 *MYCN*-amplified PDX tumor models (zccs373, COG-N-424X, COG-N-440X) were found to have similar sensitivities to BCT (IC_50_ values 145–200 ng/mL; Fig. 6A) as the four neuroblastoma cell lines from Fig. 4D (IC_50_ values 136–171 ng/mL). In a subcutaneous xenograft model of COG-N-424X in NSG mice (Fig. 6B–C), BCT-100 monotherapy delayed tumor growth and significantly extended median survival from 7.9 to 14.5 days. Addition of BCT-100 to irinotecan/temozolomide further extended median survival (23.4 days) compared to chemotherapy (18.7 days) or BCT-100 alone. Endpoint plasma arginine levels were suppressed, while liver arginine levels were unchanged (Fig. 6D). Although not reaching statistical significance due to the large variance, tumor arginine levels were lower in BCT-100 monotherapy and combination treated mice. Similar extension of survival by BCT-100 monotherapy (15.6 days vehicle vs. 20.2 days BCT-100) and potentiation of irinotecan/temozolomide chemotherapy (23.8 days irinotecan/temozolomide vs. 30.7 days irinotecan/temozolomide/BCT-100) was also observed for a second PDX xenograft model COG-N-440X (Fig. 6E–F). BCT-100 treatment was thus effective in delaying tumor growth, prolonging survival, and enhancing the anti-tumor effect of chemotherapy across transgenic, cell line xenograft, and patient-derived xenograft models of neuroblastoma.

## Discussion

Here, we provide evidence that neuroblastoma cells are sensitive to the pegylated human recombinant arginase BCT-100, with arginine depletion halting cell proliferation, decreasing global protein synthesis, and extending the survival of neuroblastoma-bearing mice as a monotherapy and in combination with standard-of-care chemo-immuno-therapy.

Adaptive responses to arginine depletion are context dependent. Cell cycle arrest has been observed for AML, hepatocellular carcinoma, and melanoma cells treated with BCT-100 [37–39], paediatric cancer cell lines treated with arginine deiminase [40], and various cancer cells cultured in arginine-free media [41, 42]. Although cell cycle arrest was not directly assessed in this study, it would account for the proliferation block we observed in neuroblastoma cell lines. Progression to cell death via apoptosis often occurs with prolonged arginine deprivation [7, 42–46]. We observed a reduction in cell number with sustained BCT-100 treatment, however proliferation was recoverable upon BCT-100 withdrawal and replenishment of arginine in the culture media, indicating the presence of a resistant subpopulation. This prolonged survival under BCT-100 treatment mirrors findings in breast, lung, and ovarian cell lines cultured with arginase I or in arginine-free media [42, 47] as well as in sarcoma and melanoma cell lines treated with ADI-PEG 20 [48, 49]. The heterogeneity of response underscores the complexity and context-dependent nature of arginine resistance mechanisms and highlights the importance of combination treatment strategies when employing arginine-depleting therapies.

We observed effects on two primary arginine level sensing mechanisms following BCT-100 treatment, namely the mTORC1 pathway through decreased phosphorylation of ribosomal S6, and the GCN2 pathway through strongly induced expression of the integrated stress response protein ATF4, downstream of GCN2 and eIF2α. While we did not observe increased eIF2α phosphorylation [33], the absence of eIF2α phosphorylation upon arginine starvation has also been observed for multiple myeloma and breast cancer cells [35, 50], possible through the phosphatase GADD34, a downstream target of ATF4 that restores protein synthesis by dephosphorylating eIF2α in a feedback mechanism [51]. Indeed, this adaptation may allow continued proliferation under nutrient-limiting conditions.

Despite induction of ATF4 and subsequently ASNS following BCT-100 treatment, the neuroblastoma cell lines did not show evidence of aspartate exhaustion, reduced expression of mitochondrial electron transport chain complex proteins, or mitochondrial dysfunction, as has been described for more genetically complex adult cancers [35, 52–54]. It remains to be determined whether this differential mitochondrial susceptibility reflects underlying cancer subtype- or cell line-specific differences in mitochondrial fitness.

Resistance to arginine starvation invariably involves adaptations that enable alternative sourcing of arginine, or that prolong survival until arginine becomes available. While ASS1 expression did not correlate with BCT-100 IC_50_ in neuroblastoma cell lines, upregulation of ASS1/ASL/OTC was observed in some BCT-100 treated Th-*MYCN* tumors. Additionally, evidence of autophagy was seen in at least one cell line and inhibition of autophagy in this line modestly enhanced sensitivity to BCT-100, consistent with studies of arginine-depleting enzymes in other solid tumors [43, 53, 55, 56]. However, the extent to which autophagy and other arginine-scavenging mechanisms such as macropinocytosis [57–59] mediate resistance to BCT-100 treatment, particularly in an in vivo context, needs further exploration.

Synthetic lethalities and collateral sensitivities offer additional opportunities to leverage arginine deprivation as a therapeutic. Upon ADI-PEG 20 treatment, leiomyosarcoma and melanoma cells shunted glucose-derived carbons into the serine biosynthesis pathway, leading to susceptibility to anti-folates and glutaminase inhibition [54]. Similarly, PHGDH inhibition synergized with ADI-PEG 20 in neuroblastoma cell lines and xenografts [6]. In this study, BCT-100 treatment reduced glycolysis and cholesterol/lipid metabolism pathway enrichment, which could reflect reduced mTORC1 activity and ISR activation, given that both pathways are known to regulate glycolytic and lipid metabolism genes [60, 61]. This BCT-100 induced glycolytic and lipid metabolism alteration is consistent with other studies of ADI-PEG 20 and arginine starvation [54, 62] and indeed, elevated glycolytic activities were found in ADI-PEG 20-resistant melanoma cell lines [63]. Metabolite profiling of BCT-100 treated neuroblastomas may therefore provide clues for further combinatory therapeutic opportunities.

The anti-proliferative effect of BCT-100 was potentiated in combination with irinotecan/temozolomide in vivo. Synergy between arginine depletion and DNA damaging agents is not unexpected given that arginine can be used to generate aspartate for nucleotide synthesis; polyamines, which modulate chromatin structure and DNA repair enzymes; and S-adenosylmethionine for histone and DNA methylation. Other DNA-targeting or anti-microtubule agents used to treat high-risk neuroblastoma therefore might also synergize with BCT-100, given the potential for both to create genomic instability and elevate ROS.

While there are limited clinical trials where an arginine-depleting enzyme has been combined with a chemotherapeutic agent, the phase I studies in adults with advanced solid tumors have invariably shown early evidence of clinical activity with no additional dose-limiting toxicities [64–66]. Furthermore, ADI-PEG 20 in combination with pemetrexed plus cisplatin or carboplatin showed positive Phase III trial results in adult subjects with pleural mesothelioma, with improvements in overall and progression free survival [67]. As BCT-100 monotherapy was well-tolerated in a pediatric population [10], it is highly promising that we observed potentiated cytotoxicity of BCT-100 with standard-of-care chemotherapeutics without increased toxicity.

In addition to potentiation with chemotherapy, we observed enhanced activity upon addition of BCT-100 to anti-GD2 immunotherapy. While NK cell proliferation and IFNγ secretion are severely impacted in arginine-free environments, there are conflicting findings described for the influence of low arginine on NK cell viability and cytotoxic functions [68, 69], though notably NK cells required 10-fold less arginine than T cells to maintain half-maximal proliferation [68]. The ability of NK cells to better tolerate low-arginine conditions and the arginine-independency of complement-dependent cytotoxicity may account for the continued activity of the monoclonal antibody therapy observed under arginine-deprived conditions in this study. Given the need to balance the benefit of targeting arginine auxotrophic tumors *via* arginine depletion with adverse effect of this depletion on the function of immune cells, though, future studies should examine the tumor immune microenvironment in patient tumors treated with BCT-100.

## Conclusions

The need for novel therapeutic agents that are non-genotoxic and effective against high-risk neuroblastoma is urgent. The arginase BCT-100 can potentiate standard-of-care chemotherapy and anti-GD2 immunotherapy in preclinical models of high-risk neuroblastoma without increasing toxicities, providing a rationale for clinical investigation of BCT-100 combinations therapy.

## Supplementary Information

Below is the link to the electronic supplementary material.

Supplementary Material 1: Figure S1. BCT-100 reduces global protein translation and activates cell stress response

Supplementary Material 2: Figure S2. BCT-100 anti-proliferative effect is not rescued by aspartate supplementation

Supplementary Material 3: Figure S3. BCT-100 exposure suppresses proliferation, glycolysis, and lipid metabolism in vitro

Supplementary Material 4: Figure S4. BCT-100 exposure does not alter urea cycle enzyme or arginine importer expression in short-term cultures

Supplementary Material 5: Figure S5: BCT-100 enhances chemotherapy efficacy in vitro and alters urea cycle enzyme expression in vivo

Supplementary Material 6: Figure captions

## Data Availability

The Sequencing Quality Control (SEQC) consortium and Neuroblastoma Research Consortium (NRC) primary neuroblastoma datasets analysed during the current study are available in the R2 Genomics Analysis and Visualization Platform (http://r2.amc.nl) and Gene Omnibus repository (GEO accession numbers GSE62564 and GSE85047 respectively). The proteomics dataset generated during the current study is available from the corresponding author on reasonable request. ZERO PDX models are available upon request from the Children’s Cancer Institute Tumour Bank.

## Abbreviations

ADI-PEG 20: Pegylated arginine deiminase
BCT-100: Pegylated recombinant human arginase I
fGSEA: Fast gene set enrichment analysis
INSS: International Neuroblastoma Staging System
IP: Intraperitoneally
LC-MS/MS: Liquid chromatography tandem mass spectrometry
PDX: Patient derived xenograft
ssGSEA: Single sample gene set enrichment analysis
