# Supplementary figures and images for: Arginine depletion potentiates standard-of-care chemo-immunotherapy in preclinical models of high-risk neuroblastoma
Source: J Exp Clin Cancer Res. 2025 Aug 14;44:239. doi: 10.1186/s13046-025-03502-8 (PMC12351974; doi:10.1186/s13046-025-03502-8)

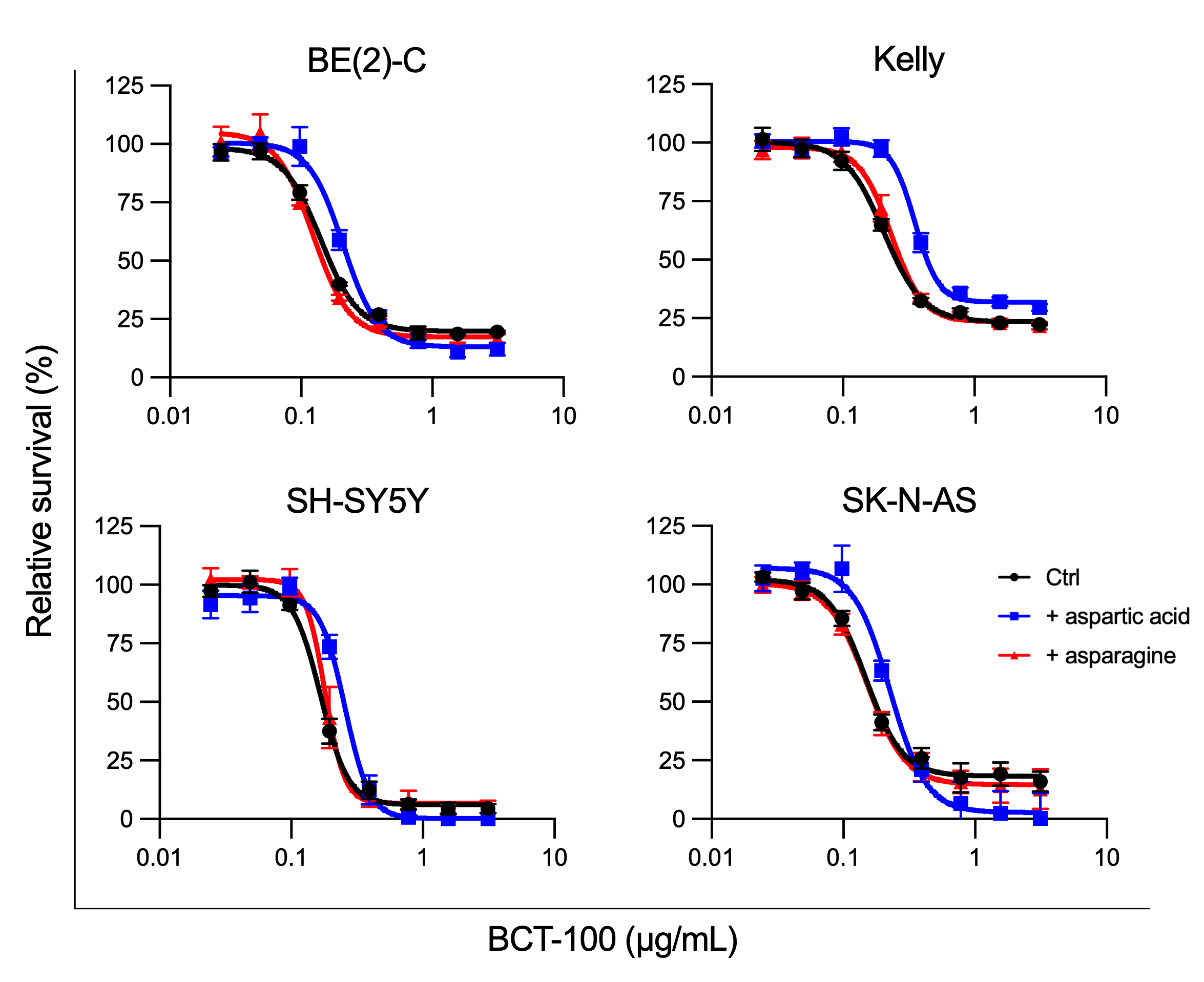

Supplement: Supplementary file 1 — Supplementary Material 1: Figure S1. BCT-100 reduces global protein translation and activates cell stress response [file 13046_2025_3502_MOESM1_ESM.tiff]

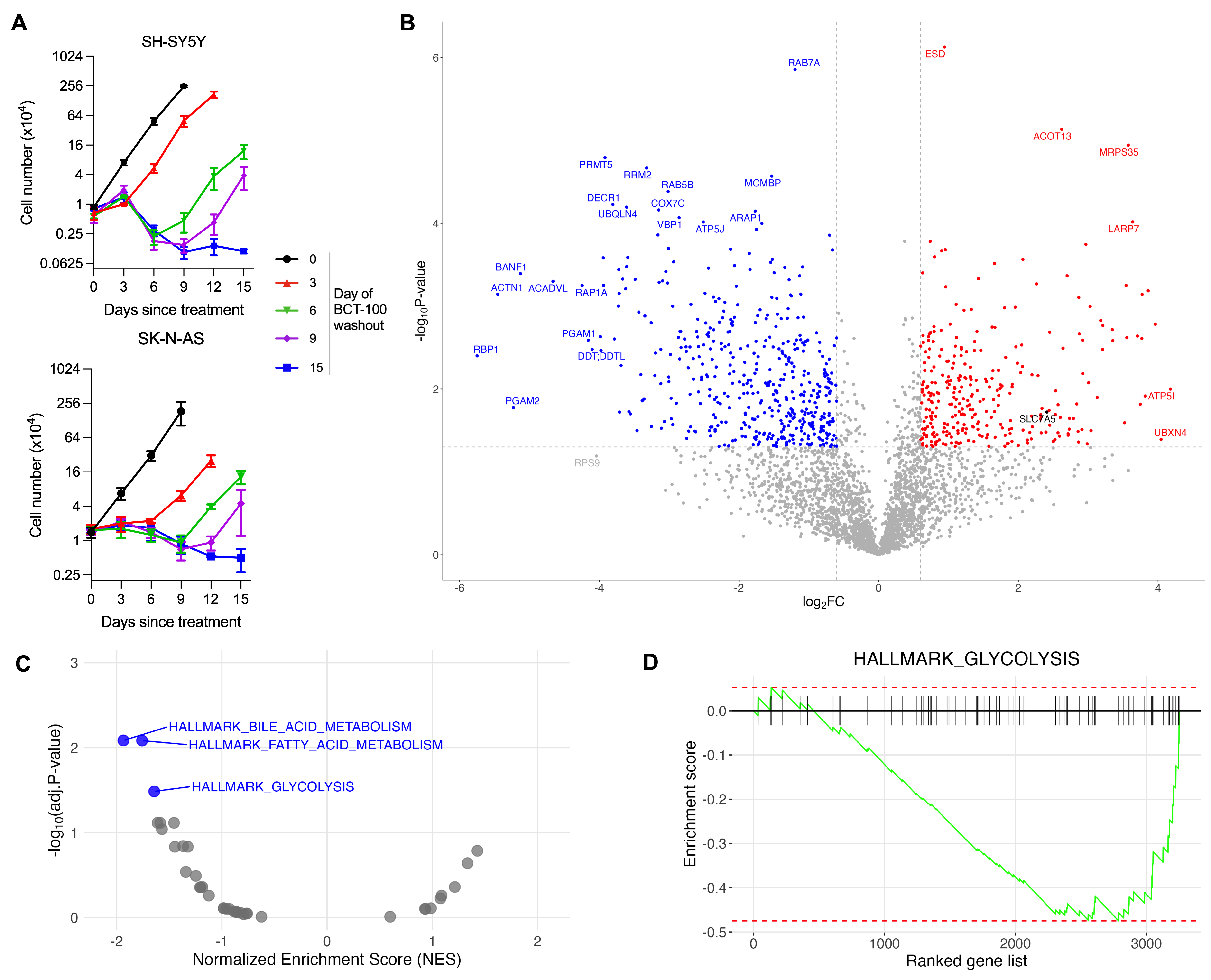

Supplement: Supplementary file 2 — Supplementary Material 2: Figure S2. BCT-100 anti-proliferative effect is not rescued by aspartate supplementation [file 13046_2025_3502_MOESM2_ESM.tiff]

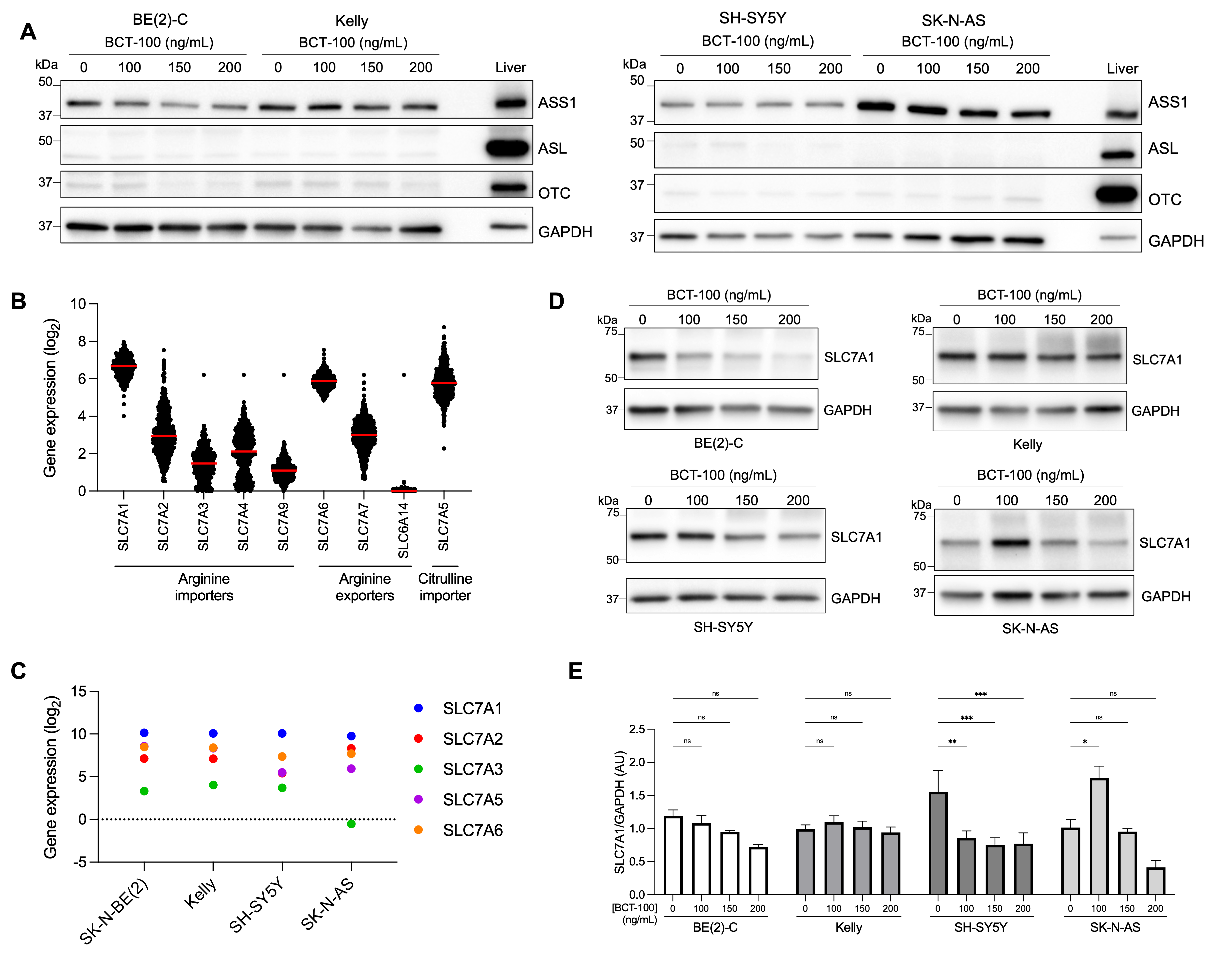

Supplement: Supplementary file 3 — Supplementary Material 3: Figure S3. BCT-100 exposure suppresses proliferation, glycolysis, and lipid metabolism in vitro [file 13046_2025_3502_MOESM3_ESM.tiff]

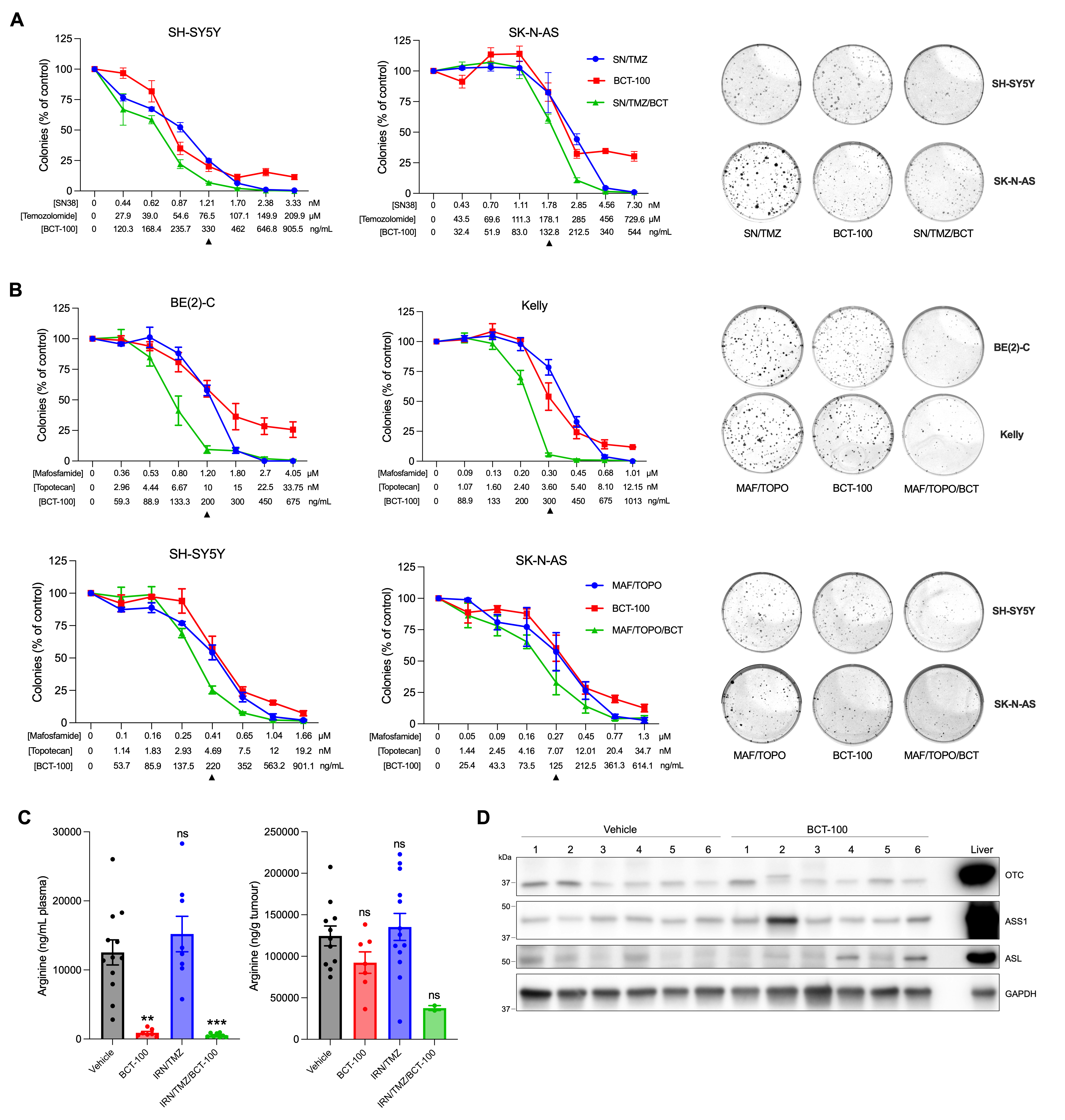

Supplement: Supplementary file 4 — Supplementary Material 4: Figure S4. BCT-100 exposure does not alter urea cycle enzyme or arginine importer expression in short-term cultures [file 13046_2025_3502_MOESM4_ESM.tiff]

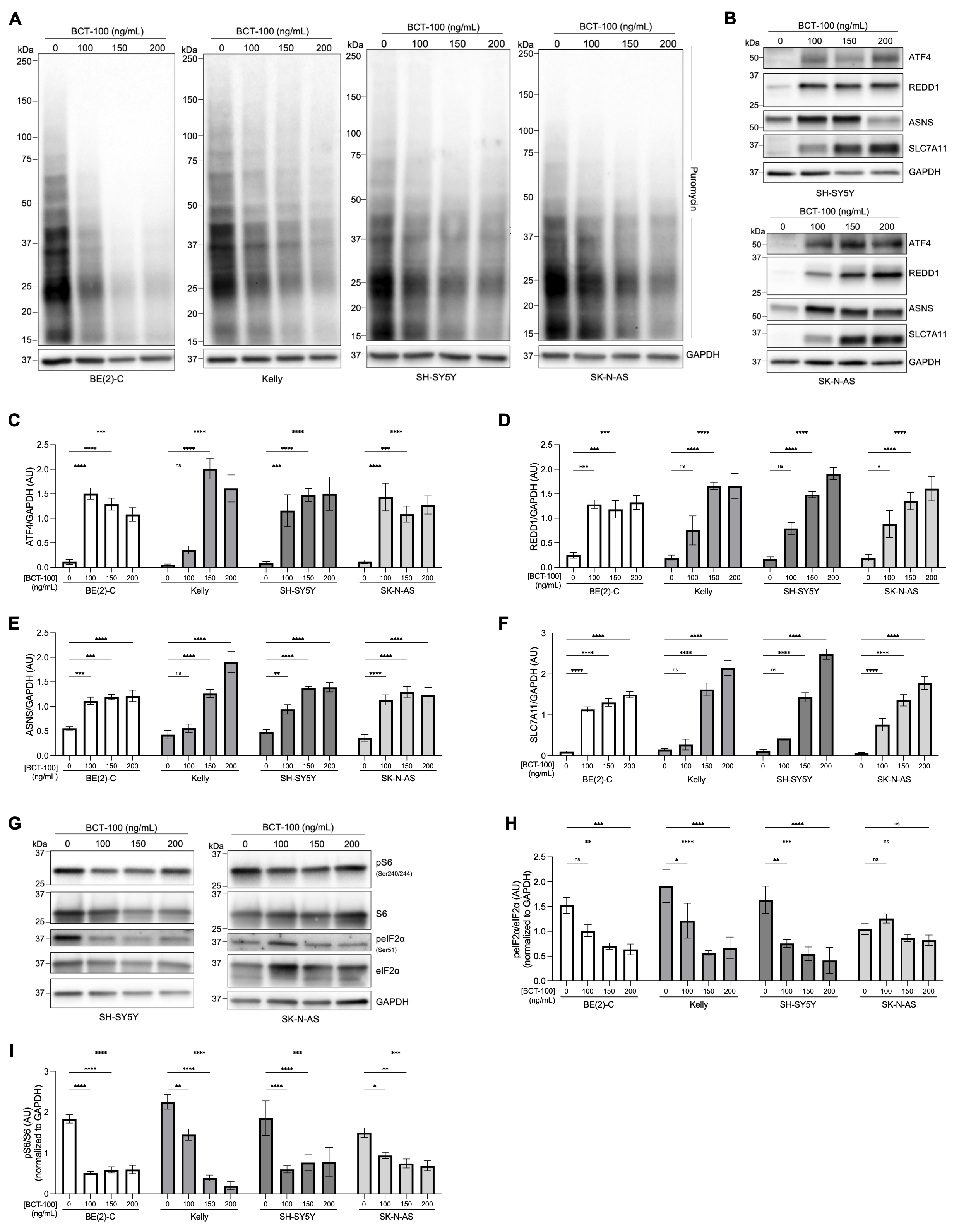

Supplement: Supplementary file 5 — Supplementary Material 5: Figure S5: BCT-100 enhances chemotherapy efficacy in vitro and alters urea cycle enzyme expression in vivo [file 13046_2025_3502_MOESM5_ESM.tiff]
