# Supplementary material for: Arginine depletion potentiates standard-of-care chemo-immunotherapy in preclinical models of high-risk neuroblastoma
Source: J Exp Clin Cancer Res. 2025 Aug 14;44:239. doi: 10.1186/s13046-025-03502-8 (PMC12351974; doi:10.1186/s13046-025-03502-8)
Supplement: Supplementary file 6 — Supplementary Material 6: Figure captions [file 13046_2025_3502_MOESM6_ESM.docx]

**Figure Captions**

Supplementary Material 1: Figure S1. BCT-100 reduces global protein translation and activates cell stress response.

Supplementary Material 2: Figure S2. BCT-100 anti-proliferative effect is not rescued by aspartate supplementation.

Supplementary Material 3: Figure S3. BCT-100 exposure suppresses proliferation, glycolysis, and lipid metabolism in vitro.

Supplementary Material 4: Figure S4. BCT-100 exposure does not alter urea cycle enzyme or arginine importer expression in short-term cultures.

Supplementary Material 5: Figure S5: BCT-100 enhances chemotherapy efficacy in vitro and alters urea cycle enzyme expression in vivo.
